# Timing and rate of nitrogen fertilization influence maize yield and nitrogen use efficiency

**DOI:** 10.1371/journal.pone.0233674

**Published:** 2020-05-29

**Authors:** Benjamin Davies, Jeffrey A. Coulter, Paulo H. Pagliari

**Affiliations:** 1 Department of Soil, Water and Climate, University of Minnesota-Twin Cities, St. Paul, Minnesota, United States of America; 2 Department of Agronomy and Plant Genetics, University of Minnesota-Twin Cities, St. Paul, Minnesota, United States of America; Harran University, TURKEY

## Abstract

Timing and rate of nitrogen (N) fertilizer application can influence maize (*Zea mays* L.) grain yield, N uptake, and nitrogen use efficiency (NUE) parameters, but results have been inconsistent across the upper Midwest. This study compared single (fall and preplant) and split applications of differing N rates for maize under irrigated conditions on loamy sand at Becker, MN and under rainfed conditions on loam and clay loam soils at Lamberton, MN and Waseca, MN, respectively, in 2014 to 2016. Fall and preplant applications of N were applied at recommended and 125% of recommended rates (RN) according to University of Minnesota guidelines. Split-application treatments included a two-way (Sp, applied at 75% and 100% of RN) and a three-way split (TSp applied at 50%, 75%, and 100% of RN), with the total N rate equally split among application times. At Becker, maize grain yield with TSp was 12.6 to 15.7 Mg ha^-1^ among years and significantly greater than that with fall or preplant treatments. The TSp treatment also improved agronomic efficiency (AE) and recovery efficiency (RE) by an average of 30% over fall or preplant treatments. At Lamberton, maize grain yield, AE and RE did not differ among treatments. However, TSp75 improved AE by 8.3 kg kg^-1^ while producing comparable yields to fall and preplant treatments. At Waseca, Sp or TSp improved grain yield and AE compared with fall treatments. These results suggest that split applications of N can increase maize grain yield, AE, and RE on irrigated coarse-textured soils and applying N fertilizer near planting or as a split application can improve N management on non-irrigated clay loam soils.

## Introduction

The rate and timing of N application are important management decisions for maize (*Zea mays* L.) production. The goal of N management is to minimize loss and maximize crop uptake [1]. A survey of nearly 1500 maize growers in Minnesota found that 33% of growers applied N fertilizer in the fall, 59% of growers applied N fertilizer as a preplant in the spring, and only 9% of growers applied the majority of their N fertilizer as sidedress [2]. Although there can be logistical advantages and cost savings for applying N in the fall, a spring preplant application often provides greater economic return to maize production with less N loss [3,4]. Postemergence N application has been shown to increase maize grain yield compared with fall or spring application on course-textured soils and when there are excessively wet spring conditions on fine-textured soils [5, 6]. However, other studies have not found significant difference in maize grain yield between N applied near planting compared with split application [5, 7, 8]. Maize demand for N during early vegetative stages is low, after which it increases rapidly and remains high for several weeks [9]. Based on the relationship between maize N uptake and growth, there is opportunity to split the total N fertilizer input for maize into multiple applications to match N supply with maize N uptake and reduce the risk of N loss [10]. Strategies for this include a reduction in fall-applied N and greater use of N applications during the growing season [11, 12].

The effect of N fertilizer application timing on maize grain yield has produced mixed results that are often site-specific. In Iowa, there was no consistent difference in grain yield when N was applied at the two leaf-collar stage of maize or equally split between the two and six or twelve leaf-collar stages [5]. This contrasts with an earlier study where a split application of N applied to maize near the two and sixteen leaf-collar stages resulted in lower grain yield compared to a single application near the two leaf-collar stage [13]. Similarly, Venterea and Coulter [14] found that applying N at planting, or as an equal three-way split between planting, at the six leaf-collar stage, and the fourteenth leaf-collar stage did not significantly impact maize grain yield on a silt loam soil. In a different study, the split application of N on a clay loam soil increased maize grain yield by 4.5% compared with a single preplant application [15]. Similar findings were obtained from southwestern Ontario, where N applied at planting produced 11% greater maize grain yield compared with a split application with 22 and 130 kg N ha^-1^ applied at the two and six leaf-collar stages, respectively [16].

Efficient N use often results from improved N recovery due to minimal losses of N to denitrification, leaching, and volatilization [17]. Maize nitrogen use efficiency (NUE) is estimated globally to be 33%, in part due to loss of fertilizer N from leaching below the root zone, denitrification, and soil- and plant-derived volatilization [18, 19]. Low NUE in maize systems has contributed to surface water pollution in the Mississippi watershed and hypoxia in the Gulf of Mexico [20]. Results from studies assessing the impact of timing of N fertilizer application on NUE suggest that applying N fertilizer as a split or preplant application often results in improved NUE compared with fall application [17, 21]. Indices for assessing NUE include (i) agronomic efficiency (AE), the increase in grain yield per unit N applied and (ii) recovery efficiency (RE), the increase in aboveground N biomass per unit N applied [17]. Agronomic efficiency closely reflects the production impact of an applied fertilizer and relates directly to economic return making it a good short-term indicator. Typical AE levels of N for cereals ranges from 15–30 kg grain kg^-1^ N, with lower levels suggesting that changes in management could increase crop response or reduce input costs [22]. Recovery efficiency represents the difference in N accumulation in the aboveground parts of a crop between fertilized and unfertilized treatments relative to the quantity of N fertilizer applied. Recovery efficiency typically ranges from 40 to 65% for cereal crops and averages around 37% for maize [12, 22]. Lower values of RE suggest that changes in management could improve efficiency or that nutrients are accumulating in soil [22]. Often changes in NUE indices are influenced by the rate of N fertilizer applied. For most indices, lower N rate treatments will have greater NUE than higher N rate treatments because NUE indices often decline with each unit increase in N fertilizer. Wortmann et al. [17] reported that the mean RE was 70% at the lowest N rate compared to 40% at the highest N rate. In Argentina, a split application of N fertilizer at planting and at the six leaf-collar stage of maize phenological development (V6) improved NUE of maize over a single application at planting in three of the four years, particularly at lower N fertilizer rates [23]. Similarly, a split application of N increased NUE by 15–18% compared with a single application at planting in Pakistan [24]. In Minnesota, N recovery by maize averaged 47, 56, 56, and 61% for anhydrous ammonia applied in the fall without the nitrification inhibitor nitrapyrin (2-chloro-6-(trichloromethyl) pyridine), in the fall with nitrapyrin, in the spring without nitrapyrin, and in the spring with nitrapyrin, respectively [4].

The time of maximum N accumulation in maize development is dependent on available soil N when maize begins rapid vegetative growth. About one-half of total N uptake by maize occurs by the time maize biomass is about one-fourth of the maximum [9]. Enhanced synchrony between N fertilizer application and maize N uptake could allow for a reduction in total N fertilizer input while maintaining or increasing maize yield, thereby improving NUE [25]. Maize N uptake can also be influenced by soil type as demonstrated in a greenhouse study where maize tissue N concentration was always greater with silt loam soil compared with fine sand [26].

Since N can be lost from cropping systems through several pathways, a single solution to best managing N is unlikely [27]. With varied results on the impact of split-applied N fertilizer there is still a need to better understand N management strategies. Particularly how to enhance NUE in maize with regards to how maize yield and NUE indices are affected when N fertilizer is split-applied during the growing season on irrigated and rainfed soils. The objective of this study was to examine how N and timings of N fertilizer application impact maize yield and NUE on irrigated and rainfed soils in the upper Midwest. Our hypothesis was that on the irrigated coarse-textured soils, split applications of N would improve maize grain yield and NUE when compared with a single application in the fall or spring; while on rainfed medium- and fine-textured soils applying N at planting or as split-applications would improve maize yield and NUE when compared with fall application.

## Materials and methods

### Site description and experimental design

Field experiments were conducted at University of Minnesota Research and Outreach Centers near Becker, MN (45° 39’ N, 93° 89’ W), Lamberton, MN (44° 24’ N, 95° 30’ W), and Waseca, MN (44° 07’ N, 93° 52’ W) from 2014 to 2016. The soil at Becker, MN was a Hubbard-Mosford loamy sand complex (Sandy, mixed, frigid Typic Hapludolls or frigid Entic Hapludolls) with organic matter (OM) level of 13 g kg^-1^ and received sprinkler irrigation during the growing season. Soils at the rainfed sites at Lamberton and Waseca were Normania loam (Fine-loamy, mixed, superactive, mesic Aquic Hapludolls) with an OM of 23 g kg^-1^ and Nicollet clay loam (Fine-loamy, mixed, superactive, calcareous, mesic Typic Endoaquolls) with an OM of 36 g kg^-1^, respectively. The experimental design was a randomized complete block design with four replications. Plots were 4.6 m (six 76-cm rows) by 15 m at Becker and Waseca, MN, and 6.1 m (eight 76-cm rows) by 12 m at Lamberton, MN. A 76-cm row was placed between each plot. In 2013, all sites were planted to soybean [*Glycine max* L. (Merr.)] and then planted to maize from 2014 to 2016. Treatments were applied to the same plots in each year. Tillage at each location involved disking to a depth of 9 cm after soybean harvest, disk ripping to a depth of 25 cm in the fall after maize harvest, and field cultivating to a depth of 9 cm in the spring prior to planting soybean and maize. The maize cultivar used was Dekalb DKC 53–56 RIB Blend planted at 86,500 seeds ha^-1^, approximately 31 kg seed ha^-1^. Weeds were controlled using pre- and post-emergence herbicides based on University of Minnesota guidelines. Phosphorus, potassium and sulfur were applied at 78-78-22 (P_2_O_5_-K_2_O-SO_4_) kg ha^-1^ at Becker and Waseca and 112-78-22 kg ha^-1^ (P_2_O_5_-K_2_O-SO_4_) at Lamberton based on University of Minnesota guidelines [28]. Monthly precipitation was collected from three National Weather Service weather stations and compared to the 30-yr average (1981–2010). Weather stations were chosen based on proximity to study sites and availability of 30-yr data. Field irrigation records for the experiment at Becker were obtained from the farm manager.

The treatments in this study consisted of a non-N-fertilized control plus nine treatments representing different combinations of N fertilizer application timing and rate: F100, single application of SuperU in the fall at 100% of RN; F125, single application of SuperU in the fall at 125% of RN; PP100, single application of urea at planting at 100% of RN; PP125, single application of urea at planting at 125% of RN; Sp75, two-way split application of urea at planting (one-half) and V6 (one-half) at 75% of RN; Sp100, two-way split application of urea at planting (one-half) and V6 (one-half) at 100% of RN; TSp50, three-way split application of urea at planting (one-third), V6 (one-third), and silking (one-third) at 50% of RN; TSp75, three-way split application of urea at planting (one-third), V6 (one-third), and silking (one-third) applied at 75% of RN; TSp100, three-way split application of urea at planting (one-third), V6 (one-third), and silking (one-third) at 100% of RN. The N rates applied each year for the study were based on the recommended N rates from University of Minnesota guidelines [28]. These were 168 kg N ha^-1^ for Becker and 135 kg N ha^-1^ for Lamberton and Waseca for maize following soybean in 2014, and 235 kg N ha^-1^ for Becker and 202 kg N ha^-1^ for Lamberton and Waseca for maize following maize in 2015 and 2016. The source of N used for fall applications was SuperU® (46-0-0 of N-P_2_O_5_-K_2_O), a urea-based granule with dicyandiamide and N-(n-butyl)thiophosphoric triamide to inhibit nitrification and urease (Koch Agronomic Services, LLC., Kansas, USA). The source of N for preplant and in-season applications was urea (46-0-0 of N-P_2_O_5_-K_2_O).

All fertilizer applications were made by hand. Fertilizers were broadcast uniformly on the soil surface and incorporated with tillage immediately after application in early November for fall treatments and prior to planting in late April through early May. At Becker, in-season fertilizer applications were applied on the soil surface in 8-cm-wide bands centered between maize rows and incorporated with irrigation. At Lamberton and Waseca, in-season fertilizer applications were applied in 8-cm-wide by 5-cm deep furrows centered between maize-rows that were made using a hoe and closed with soil immediately after fertilizer application. Each year the V6 fertilizer treatments were applied in mid to late June, while the silking fertilizer treatments were applied in late July.

#### Aboveground nitrogen uptake

Each growing season, whole plant samples were collected from each plot approximately one week before and one week after the V6 N application (Pre- and Post-V6, respectively), three weeks after the V6 N application (3wk-V6), one week before and one week after the silking stage of maize phenological development (R1) N application (Pre- and Post-R1, respectively), three weeks after the R1 N application (3wk-R1), and at maize physiological maturity (PM). The only exception was in 2016 at Waseca, when samples were not collected at Pre-R1 due to persistent wet soil conditions.

At each sampling event, six plants representing the plot area were collected from rows two and five. In each row a plant was taken from the starting third, middle, and back third. Samples were not collected within 1.5 m of the plot edges or next to previous sampled plants. Plant samples were weighed wet in the field, then chopped, and a sub sample was collected to determine moisture content. These samples were then dried in a forced-air dryer at 60Cº until constant mass and the dry weight was determined for each treatment. The dry stover samples were then ground to 1 mm and analyzed for N concentration by combustion using a CHNS analyzer (Vario MACRO, Elementar, Langenselbold, Germany).

#### Grain yield

Maize grain yield was measured at physiological maturity. At Becker a total of 6 meters from the center of rows three and four in each plot were hand harvested and a subsample of the grain was used for determination of total nutrient uptake at harvest. Ears and stalks were separated and weighed wet in the field. The stalks were then chopped in field, and a sub sample was collected to determine moisture content along with a subsample of ears. These samples were then dried in a forced-air dryer at 60Cº until constant mass and the dry weight was determined for each treatment. The grain samples were then ground to 1mm and analyzed for N concentration by combustion using a CHNS analyzer (Vario MACRO, Elementar, Langenselbold, Germany). Yield for each plot was adjusted to 15.5% grain moisture for all treatments. At Lamberton and Waseca, MN, crop grain yield was determined at harvest using a plot combine harvesting rows three and four and a subsample of the grain was retained for grain moisture and total nutrient uptake.

Nitrogen use efficiency was calculated as AE and RE using the following equations:

$${AE}_{i}=(Y_{Ni}–Y_{0})/N_{i}$$ (1)

where *Y_Ni_* is maize grain yield for the *i^th^* N fertilizer treatment, *Y*_0_ is maize grain yield for the non-N-fertilized treatment, and *N_i_* is the *i^th^* N fertilizer treatment.

$${RE}_{i}=({UN}_{Ni}–{UN}_{0})/N_{i}$$ (2)

where *UN_Ni_* is the total N in above ground biomass at physiological maturity for the *i^th^* N fertilizer treatment, *UN*_0_ is the total N in above ground biomass at physiological maturity for the unfertilized treatment, and *N_i_* is the *i^th^* N fertilizer treatment.

#### Statistical analysis

Data were analyzed at *P* ≤ 0.05 using the GLIMMIX procedure of SAS 9.4 [29]. For grain yield, AE, and RE, location, year, and treatment were considered fixed effects, and replication was considered a random effect. When fixed effects were significant, pairwise mean comparisons were made using the lines option in the GLIMMIX procedure of SAS. For maize N uptake, sampling time and year were considered repeated measurement and the covariance structure that best fit the model for each parameter was assessed by checking the Akaike Information Criteria (AIC) among all possible covariance structures. When appropriate, pairwise mean comparisons were made at *P ≤* 0.05 using the lines option in the GLIMMIX procedure of SAS. Data were analyzed separately by location due to many interactions among location and year, treatment, and sampling time.

## Results

### Weather

#### Becker

With the ability to irrigate, any precipitation deficiencies were supplemented from June through September (Table 1). Throughout the field trials, the month of May received 17 to 100 mm more precipitation than the historical average. In 2015, a drier September required 15 mm of supplemental irrigation. Precipitation in 2016 was similar to historical averages except in July, September and October which received an additional 87, 25, and 74 mm precipitation, respectively, compared with historical averages.

**Table 1:** Monthly total precipitation during 2014 to 2016 and the 30-yr (1981–2010) average for Becker, Lamberton, and Waseca, MN.

	January	February	March	April	May	June	July	August	September	October	November	December
	Precipitation (mm)											
Becker												
30-yr average	21	19	43	68	84	113	93	102	91	65	39	27
2014	26	26	26	180	184	160+6 †	88+95	112+126	60	19	43	13
2015	11	12	9	37	149	113+53	166+109	222+39	54+15	93	87	25
2016	8	18	27	67	101	76+64	192+94	141+64	147	55	58	24
Lamberton												
30-yr average	15	18	37	71	88	104	91	80	78	52	33	19
2014	18	13	25	87	46	188	30	95	154	12	13	25
2015	11	5	10	31	139	128	96	113	87	41	84	34
2016	8	17	51	85	141	66	176	135	134	72	47	29
Waseca												
30-yr average	32	25	63	82	100	119	112	121	93	68	55	38
2014	36	40	35	142	73	329	30	81	59	35	28	18
2015	19	19	29	70	121	194	188	152	149	31	101	88
2016	11	22	49	50	95	121	227	297	376	79	41	54

†Precipitation plus irrigation

#### Lamberton

In 2014, monthly precipitation from May through August occurred in an alternating dry-wet manner throughout the growing season. September 2014 was particularly wet with 76 mm more precipitation than the historical average. In 2015, monthly precipitation at Lamberton was closer to the historical averages. 2016 was the wettest of the three years for Lamberton. A wet May, July, August, and September received 53 to 85 mm more precipitation compared with historical averages.

#### Waseca

In 2014, April received 60 mm more precipitation than historical averages for the month. The month of May was drier than historical averages; however, June was an exceptionally wet month, receiving 329 mm of precipitation, 210 mm more than historical averages. The rest of the year was drier, with July, August, September, and October receiving 82, 40, 34, and 33 mm less precipitation than historical averages, respectively. Greater than historical averages precipitation was observed in 2015 during June through September receiving an additional 32 to 76 mm more precipitation than historical averages. In 2016, precipitation was slightly lower than average from January through June; however, July, August and September received 115, 176, and 283 mm more precipitation, respectively, than the historical averages for each month.

### Tests of fixed effects

There was a significant location × year × treatment interaction for maize grain yield and agronomic efficiency (Table 2). While maize RE for applied N fertilizer was significantly affected by treatment × location, year × treatment, and year × location interactions. For maize nitrogen uptake, there was a three-way interaction of treatment × time of sampling × year at Becker and Waseca, while at Lamberton there was a two-way interaction of treatment × time of sampling, treatment × year, and year × time of sampling (Table 3).

**Table 2:** Tests of fixed effects on maize grain yield, agronomic efficiency (AE), and recovery efficiency (RE).

Fixed effect	Yield	AE	RE
	*------------------------P > F------------------------*		
Location	< .0001	< .0001	< .0001
Year	< .0001	< .0001	< .0001
Treatment	< .0001	< .0001	< .0001
Location × year	< .0001	< .0001	< .0001
Location × treatment	< .0001	< .0001	0.0002
Year × treatment	< .0001	0.007	0.032
Location × year × treatment	< .0001	< .0001	0.558

**Table 3:** Tests of fixed effects on maize nitrogen uptake.

Fixed Effect	N		
	Becker	Lamberton	Waseca
	*-------------------P > F-------------------*		
Year	< .0001	< .0001	< .0001
Treatment	< .0001	< .0001	< .0001
Time of Sampling	< .0001	< .0001	< .0001
Year × treatment	< .0001	0.0004	< .0001
Treatment × time of sampling	< .0001	< .0001	< .0001
Year × time of sampling	< .0001	< .0001	< .0001
Treatment × time of sampling × year	0.0001	0.196	< .0001

### Maize grain yield

#### Becker

In 2014, maize grain yield for TSp100 was 12.6 Mg ha^-1^, 21 to 123% greater than the other treatments (Table 4). The other split application treatments also produced greater grain yield than all PP and F treatments regardless of N rate. Given that Becker is a coarse-textured soil, it is likely that the high amount of precipitation in April through June in 2014 resulted in leaching of N applied with the F and PP below the maize root zone (Table 1). In 2015, the highest-yielding treatments at Becker were Sp100 and TSp100, producing 15.2 and 14.5 Mg ha^-1^ of grain, respectively, an increase of 12 to 210% compared with other treatments (Table 4). Yield was not significantly different among PP125, Sp75 and TSp75 despite a 40% difference in N rate. Fall application of N, regardless of rate, yielded lower than any PP, Sp or TSp treatments regardless of rate. Similar to 2014, high precipitation in May 2015 likely led to N leaching in F treatments compared with other treatments. In 2016, TSp100 yielded more grain compared with F100, F125, PP100, Sp75, TSp50, and TSp75. Both Sp100 and PP125 produced more grain than TSp50, PP100, and F applications.

**Table 4:** Maize grain yield as affected by treatment, location, and year.

	Becker						Lamberton						Waseca					
Treatment†	2014		2015		2016		2014		2015		2016		2014		2015		2016	
	Mg ha^-1^																	
C	6.0	e‡	4.9	e	4.9	g	9.5	e	5.0	d	6.3	d	4.5	g	6.9	d	6.1	d
F100	5.7	e	6.8	d	8.0	f	13.8	abc	13.5	a	13.9	ab	8.6	de	15.3	a	11.3	bc
F125	5.8	e	8.2	d	10.2	e	14.5	ab	14.2	a	15.5	a	9.8	bc	14.2	ab	12.1	abc
PP100	7.1	d	10.1	c	12.6	cd	14.4	ab	13.6	a	14.5	ab	10.3	ab	13.5	b	13.4	a
PP125	7.6	d	11.7	b	14.4	ab	14.7	a	14.2	a	14.9	ab	11.2	a	14.0	ab	13.6	a
Sp75	8.9	c	12.0	b	13.6	bc	13.2	bcd	12.0	bc	14.0	ab	9.2	cd	14.0	ab	12.9	ab
Sp100	10.0	bc	15.2	a	14.7	ab	13.9	abc	13.6	a	14.9	ab	9.9	abc	15.3	a	13.0	a
TSp50	9.8	bc	9.9	c	11.1	de	12.2	d	11.0	c	11.3	c	6.6	f	11.6	c	10.8	c
TSp75	10.5	b	12.9	b	13.9	bc	12.8	cd	13.1	ab	13.5	b	7.9	ef	14.1	ab	12.7	ab
TSp100	12.7	a	14.5	a	15.7	a	13.2	bcd	13.3	ab	14.7	ab	9.5	bcd	15.5	a	13.3	a

† C, control no N applied; F100, N applied in the fall at 100% of the recommended rate; F125, N applied in the fall at 125% of the recommended rate; PP100, N applied at preplant in the spring at 100% of the recommended rate; PP125, N applied at preplant in the spring at 125% of the recommended rate; Sp75, half of the N applied at preplant in the spring and half at V6 at 75% of the recommended rate; Sp100, half of the N applied at preplant in the spring and half at V6 at 100% of the recommended rate; TSp50, a third of the N applied at preplant in the spring, a third at V6 and a third at R1 at 50% of the recommended rate; TSp75, a third of the N applied at preplant in the spring, a third at V6 and a third at R1 at 75% of the recommended rate; TSp100, a third of the N applied at preplant in the spring, a third at V6 and a third at R1 at 100% of the recommended rate.

‡Means within the column followed by different lowercase letters are significantly different at *P ≤* 0.05.

#### Lamberton

In 2014, maize grain yield did not differ when the same rate of N was applied in the fall, preplant, or as a split application (Table 4). The PP125 treatment produced a grain yield of 14.7 Mg ha^-1^, 55 and 11% greater than that of C and Sp75, respectively, and 11 to 20% greater than TSp50, TSp75, and TSp100. In addition, yield with the Sp75 treatment was not significantly different from F125, despite a 40% reduction in total N rate. Results in 2015 were similar to 2014, although no differences in yield occurred among the F, PP, Sp100, Tsp75, and Tsp100 treatments (Table 4). In 2016, there was no difference in yield among F, PP, Sp and Tsp100 treatments.

#### Waseca

In 2014, PP125 produced 11.2 Mg ha^-1^, an 18 to 149% increase in maize grain yield compared with F100, F125, Sp75, TSp50, TSp75, and TSp100 (Table 4). In 2014, yield was not different among PP100 and Sp100 but was on average 17% greater than F100. In contrast, Sp75 yielded 16% more than TSp75; however, there was no yield difference among Sp75 and Sp100. The precipitation in June could have enhanced maize utilization of N applied at V6 in the Sp75 treatment, while dry conditions during July may have led to delayed and reduced uptake of N applied at silking in the TSp treatment (Table 1). In 2014, yield was also influenced by N rate at Waseca for the F and TSp treatments, as F125 increased yield by 91% compared with F100, and TSp100 increased yield by 20 and 44% to TSp75 and TSp50, respectively (Table 4). In contrast, yield was not different between N rates for the PP and Sp treatments.

In 2015, splitting N rates into two or three applications produced the greatest yield at Waseca, especially for treatments with N applied at 100% of the recommended rate. Furthermore, grain yield with Sp75 and TSp75 was equivalent to that with F125 and PP125. In 2016, there were no differences in yield between F125, PP100, PP125, Sp75, Sp100, TSp75 and TSp100. Applying 75% of the recommended rate in two or three split applications produced grain yield equivalent to that with 125% of the recommended rate in a single application in the fall or spring. For example, grain yield did not differ significantly among Sp75, F125, and PP125, and grain yield with TSp50 was not different from that with F100 or F125.

### Agronomic efficiency

#### Becker

In 2014, agronomic efficiency was greater for TSp treatments compared with Sp, PP, and F treatments regardless of rate (Table 5). There were no differences between the F and PP treatments. This follows the yield response in 2014, where all split treatments, regardless of rate, yielded more than the PP and F treatments (Table 4). In 2015, the F treatments, regardless of rate, produced the lowest AE, followed by the PP treatments (Table 5). There was no difference in AE among TSp and Sp treatments, regardless of rate, but both were greater than the PP and F treatments. The ability of Sp75 and TSp75 to produce yields similar to PP125 resulted in a greater AE for split applications at reduced rates compared with PP treatments at recommended or increased rates (Table 4, Table 5). In 2016, there were no differences in AE between Sp75, TSp50, TSp75, and TSp100; however, TSp50 and TSp75 had a greater AE than Sp100 even though Sp100 yielded more grain than TSp50. Split applications at Becker, MN produced an agronomic efficiency ranging from 40 to 53 kg kg^-1^ (Table 5).

**Table 5:** Agronomic efficiency of applied N fertilizer as affected by treatment, location, and year.

	Becker						Lamberton						Waseca					
Treatment †	2014		2015		2016		2014		2015		2016		2014		2015		2016	
	kg kg^-1^§																	
C																		
F100	-2	c‡	8	c	13	e	32	ab	42	c	38	d	30	c	42	ab	26	d
F125	-1	c	11	c	18	e	30	ab	36	c	37	d	31	c	29	c	24	d
PP100	6	c	22	b	33	cd	37	ab	42	c	41	cd	43	ab	33	bc	36	bc
PP125	7	c	23	b	32	d	31	ab	36	c	34	d	40	abc	29	c	30	cd
Sp75	23	b	40	a	49	ab	37	ab	46	bc	51	a	46	a	47	a	45	ab
Sp100	23	b	44	a	42	bc	33	ab	43	c	43	abcd	40	abc	42	ab	34	c
TSp50	45	a	42	a	53	a	41	a	59	a	50	ab	31	c	47	a	47	a
TSp75	36	a	45	a	51	a	33	ab	53	ab	48	abc	33	bc	48	a	44	ab
TSp100	39	a	41	a	46	ab	28	b	41	c	42	bcd	37	abc	43	ab	36	bc

†C, control no N applied; F100, N applied in the fall at 100% of the recommended rate; F125, N applied in the fall at 125% of the recommended rate; PP100, N applied at preplant in the spring at 100% of the recommended rate; PP125, N applied at preplant in the spring at 125% of the recommended rate; Sp75, half of the N applied at preplant in the spring and half at V6 at 75% of the recommended rate; Sp100, half of the N applied at preplant in the spring and half at V6 at 100% of the recommended rate; TSp50, a third of the N applied at preplant in the spring, a third at V6 and a third at R1 at 50% of the recommended rate; TSp75, a third of the N applied at preplant in the spring, a third at V6 and a third at R1 at 75% of the recommended rate; TSp100, a third of the N applied at preplant in the spring, a third at V6 and a third at R1 at 100% of the recommended rate.

‡Means within the column followed by different low case letters are significantly different at *p ≤* 0.05

#### Lamberton

In 2014, there was no difference in AE among treatments receiving at least 75% of the recommended N fertilizer rate. The lack of difference in AE among treatments receiving recommended rates can be attributed to similar grain yield among these treatments (Table 4). Only TSp50 had a greater AE than TSp100 (Table 5). In 2015, TSp50 had a greater AE over all other treatments except TSp75. Similarly, TSp75 had greater AE than all other treatments except Sp75. There was no difference in AE among TSp100 and F, PP, and Sp treatments. Given that grain yield with TSp75 was similar to that with N applied at 100 or 125% of recommended rates, the greater AE was expected. Results were similar in 2016, where both Sp75 and TSp50 had a greater AE than F, PP treatments regardless of rate and Sp75 also had a greater AE than TSp100. Given that yields in these treatments did not differ in Lamberton, lower N rates produced greater AE. For treatments receiving recommended rates of N fertilizer, the average AE ranged from 33 to 42 kg kg^-1^ per year, while treatments receiving less than recommended rates produced an average agronomic efficiency ranging from 37 to 53 kg kg^-1^. The high agronomic efficiency at Lamberton suggests that high yielding and efficient production is achievable.

#### Waseca

In 2014, Sp75 had an AE of 46 kg kg^-1^, greater than all F treatments, TSp50, and TSp75 (Table 5). There was no difference among PP and Sp treatments and TSp100. Surprisingly, there was no difference in AE between F100, F125, PP125 and TSp50. This is likely due to TSp50 yielding 6.6 Mg ha^-1^ in 2014 while F100, F125, and PP125 yielded 8.6, 9.6, and 11.2 Mg ha^-1^, respectively (Table 4). Given that TSp50 tends to produce greater AE due to the lower amount of fertilizer applied, the similarity in AE between TSp50 and F100, F125, and PP125 can be attributed to TSp50’s grain yield. In 2015, there was no difference in AE among F100 and all Sp and TSp treatments, regardless of rate and timing of application (Table 5). There were also no differences in AE between treatments receiving recommended rates, regardless of timing of application. This is likely due to the similar yields produced by F100 and among Sp or TSp treatments (Table 4). In 2016, there was no difference in AE between treatments receiving less than 100% recommended rates (Table 5). Fall applied treatments, regardless of rate, had an AE lower than all other treatments due to differences in yield. Among PP100, Sp100, and TSp100 there was no difference in AE. Results in 2016 suggest that the application of N applied as PP, Sp, and TSp can increase AE. Similarly, treatments receiving recommended rates (F100, PP100, Sp100, and TSp100) produced an annual mean AE ranging from 33 to 40 kg kg^-1^ per year, while treatments receiving 50 to 75% of recommended rates produced a mean AE of 37 to 47 kg kg^-1^ per year.

### In season maize nutrient uptake

#### Becker

Differences in maize tissue N uptake in most cases started around the 3wk-V6 stage and become more evident as the season progressed (Table 6). At the early developmental stages 3wk-V6 to Pre-R1, particularly 2015 and 2016, Sp treatments tended to have greater tissue N uptake compared with other treatments. At the later developmental stage from Post-R1 to PM, N uptake was greater in plots receiving Tsp treatments. It is also evident that there was a response to the N application rates. In general, the greater the N application rate the greater the N uptake by maize at Becker, for example in 2015 N uptake was 88, 114, and 135 kg for TSp50, TSp75, and TSp100, respectively. Application of N in the fall or spring had the lowest N uptake by maize, and in many instances uptake in those treatments was not different than the uptake observed in the control plots.

**Table 6:** Maize tissue N uptake at Becker 2014–2016.

Treatment	2014						
	Pre-V6†	Post-V6	3wk-V6	Pre-R1	Post-R1	3wk-R1	PM
	kg N ha^-1^						
C‡	2	15	22 bc §	29 ef	38 d	53 f	46 d
F100	2.6	16	23 bc	34 def	41 d	54 f	45 d
F125	2.5	16	21 c	31 ef	39 d	67 ef	46 d
PP100	3.1	21	31 bc	27 f	49 d	61 ef	64 cd
PP125	3.7	25	44 abc	47 cdef	56 cd	84 de	82 bc
Sp75	2.5	22	44 abc	73 ab	84 b	100 cd	68 bcd
Sp100	2.4	22	57 a	83 a	99 ab	127 b	86 bc
TSp50	2.3	19	39 abc	53 bcde	77 bc	124 bc	88 b
TSp75	2.4	19	40 abc	57 bcd	95 ab	167 a	88 bcd
TSp100	2.8	22	45 ab	69 abc	112 a	160 a	132 a
	2015						
Treatment	Pre-V6	Post-V6	3wk-V6	Pre-R1	Post-R1	3wk-R1	PM
	kg N ha^-1^						
C	0.5	13 c	23 d	35 d	35 f	54 f	39 f
F100	1.7	33 abc	52 c	55 cd	66 e	92 e	66 e
F125	2.8	41 ab	79 b	101 b	115 bc	150 bc	103 bcd
PP100	2.6	49 a	84 b	111 b	98 cd	135 cd	79 de
PP125	2.4	43 ab	77 b	99 b	103 cd	129 cd	91 cde
Sp75	1.4	28 abc	101 ab	102 b	114 bc	173 b	117 ab
Sp100	2.1	34 abc	116 a	139 a	163 a	210 a	121 ab
TSp50	1.2	20 bc	51 c	66 c	80 de	124 d	88 de
TSp75	1.6	31 abc	84 b	102 b	119 bc	205 a	114 abc
TSp100	1.5	32 abc	95 ab	109 b	137 b	220 a	135 a
	2016						
Treatment	Pre-V6	Post-V6	3wk-V6	Pre-R1	Post-R1	3wk-R1	PM
	kg N ha^-1^						
C	1.7	14 c	23 f	51 d	31 g	50 f	43 f
F100	4.9	33 bc	44 ef	113 c	65 f	80 e	62 ef
F125	4.5	39 ab	59 de	119 c	86 ef	102 e	76 e
PP100	6.7	53 ab	95 abc	170 ab	149 ab	165 bc	124 bcd
PP125	8.8	61 a	108 ab	178 ab	131 bc	176 abc	133 abcd
Sp75	4.3	45 ab	105 ab	158 b	148 ab	153 cd	121 cd
Sp100	4.1	47 ab	117 a	180 ab	156 a	169 bc	145 ab
TSp50	3.2	36 bc	76 cd	123 c	101 de	140 d	144 abc
TSp75	4.3	39 ab	85 bc	164 b	113 cd	179 ab	118 d
TSp100	5.1	53 ab	104 ab	189 a	171 a	197 a	150 a

**†**Pre-V6, soil samples taken 1 week before V6 urea applications; Post-V6, soil samples taken 1 week after V6 urea applications; 3wk-V6, soil samples taken 3 weeks after V6 urea applications; Pre-R1, soil samples taken 1 week before R1 urea application; Post-R1, soil samples taken 1 week after R1 urea applications; 3 wks-R1, soil samples taken 3 weeks after R1 urea applications; PM, soil samples taken at physiological maturity.

‡C, control no N applied; F100, N applied in the fall at 100% of the recommended rate; F125, N applied in the fall at 125% of the recommended rate; PP100, N applied at pre-plant in the spring at 100% of the recommended rate; PP125, N applied at pre-plant in the spring at 125% of the recommended rate; Sp75, half of the N applied at pre-plant in the spring and half at V6 at 75% of the recommended rate; Sp100, half of the N applied at pre-plant in the spring and half at V6 at 100% of the recommended rate; TSp50, a third of the N applied at pre-plant in the spring, a third at V6 and a third at R1 at 50% of the recommended rate; TSp75, a third of the N applied at pre-plant in the spring, a third at V6 and a third at R1 at 75% of the recommended rate; TSp100, a third of the N applied at pre-plant in the spring, a third at V6 and a third at R1 at 100% of the recommended rate.

§Means within the column followed by different lower case letters are significantly different at *p ≤* 0.05.

#### Lamberton

*Treatment × time of sampling*. Maize N uptake was affected by how N was applied prior to planting (Table 7). Differences between treatments began at the Post-V6 sampling date where maize N uptake in the F125 treatment was greater than in the control and all split applications, regardless of rate until the final sampling. At the 3wk-V6 and Pre-R1 sampling dates, the F125 treatment had taken up significantly more N than C, F100, and the Sp and TSp treatments. For much of the later season, N uptake was greater when fertilizer was applied as F125 or PP125, while N uptake in Sp and TSp treatments was intermediate. At the last sampling, PM, N uptake was similar among treatments receiving similar N fertilizer rates, regardless of time of application. In addition, there was a clear N uptake response to N rate starting at the Post-R1 and remaining consistent until PM for the Sp and TSp treatments.

**Table 7:** Treatment × year and treatment × time of sampling for maize tissue N uptake at Lamberton 2014–2016.

Treatment	2014		2015		2016		Pre-V6‡	Post-V6		3wk-V6		Pre-R1		Post-R1		3wk-R1		PM	
	kg ha^-1^																		
C†	71	e§	54	f	42	f	2.3	22	f	44	f	62	f	68	f	106	f	83	e
F100	153	a	144	bc	114	c	6.4	65	abc	118	bc	155	bc	175	bc	247	b	194	b
F125	155	a	163	a	139	a	6.6	74	a	135	a	180	a	186	ab	268	a	216	a
PP100	153	a	141	bc	129	ab	5.4	62	abcd	127	ab	171	ab	181	b	249	b	193	b
PP125	157	a	150	ab	120	bc	5.5	68	ab	121	ab	169	ab	200	a	231	bc	202	ab
Sp75	119	bc	130	cd	107	cd	4.5	47	de	101	cd	145	cd	159	cd	208	d	169	c
Sp100	134	b	143	bc	130	ab	5	56	bcd	115	bc	156	bc	183	ab	239	bc	195	b
TSp50	99	d	86	e	77	e	3.7	35	ef	78	e	99	e	117	e	144	e	137	d
TSp75	111	cd	125	d	94	d	5.4	45	de	89	de	112	e	151	d	202	d	167	c
TSp100	117	c	143	bc	111	c	4.8	50	cde	97	d	128	d	163	cd	226	c	197	b

†C, control no N applied; F100, N applied in the fall at 100% of the recommended rate; F125, N applied in the fall at 125% of the recommended rate; PP100, N applied at pre-plant in the spring at 100% of the recommended rate; PP125, N applied at pre-plant in the spring at 125% of the recommended rate; Sp75, half of the N applied at pre-plant in the spring and half at V6 at 75% of the recommended rate; Sp100, half of the N applied at pre-plant in the spring and half at V6 at 100% of the recommended rate; TSp50, a third of the N applied at pre-plant in the spring, a third at V6 and a third at R1 at 50% of the recommended rate; TSp75, a third of the N applied at pre-plant in the spring, a third at V6 and a third at R1 at 75% of the recommended rate; TSp100, a third of the N applied at pre-plant in the spring, a third at V6 and a third at R1 at 100% of the recommended rate.

‡Pre-V6, soil samples taken 1 week before V6 urea applications; Post-V6, soil samples taken 1 week after V6 urea applications; 3wk-V6, soil samples taken 3 weeks after V6 urea applications; Pre-R1, soil samples taken 1 week before R1 urea application; Post-R1, soil samples taken 1 week after R1 urea applications; 3 wks-R1, soil samples taken 3 weeks after R1 urea applications; PM, soil samples taken at physiological maturity.

§Means within the column followed by different lower case letters are significantly different at the *p ≤* 0.0

*Treatment × year*. In 2014, plant N uptake in F and PP treatments averaged between 153 to 157 kg N ha^-1^, greater than Sp and TSp treatments (Table 7). In 2015, F125 had greater plant N uptake than all treatments except PP125. There was no difference in N uptake among treatments receiving similar N fertilizer rates. In 2016, F125 had great N uptake than all treatments except PP100 and Sp100.

*Year × time of sampling*. Maize N biomass increased every sampling date until 3wk-R1 where it plateaued in 2014 and declined in 2015 and 2016 (Table 8). It is likely that the decline in maize tissue N uptake observed in 2015 and 2016 at PM was due to a significant amount of N being translocated to the developing ear. After silking, N incorporated at the beginning of stalk elongation and stored in vegetative organs has been demonstrated through ^15^N isotopes to almost totally remobilize either directly to the kernel or after transitory storage in the cob, husk, and shank [30]. The results suggest that the maize plants started to translocate nutrients into the grain sooner in the growing cycle as the study went on. In 2014, the amount of N in the plant tissue was similar between 3wk-R1 and PM, in contrast it had decreased by 30 kg N ha^-1^ in 2015, and by 83 kg N ha^-1^ in 2016. Although the reasons for this is not yet understood it could be related to physiological changes due to monocropping or be weather related. Precipitation did increase from May through September from 512 mm in 2014 to 651 mm in 2016. The increased precipitation might have impacted root growth, reducing the amount of N taken up, thus beginning the translocation of N to the cob earlier in the season.

**Table 8:** Year × time of sampling for maize tissue N uptake at Lamberton 2014–2016.

Time of sampling	2014		2015		2016	
	kg ha^-1^					
Pre-V6‡	7	m§	5	m	3	m
Post-V6	68	j	53	k	36	l
3wk-V6	119	g	105	h	83	i
Pre-R1	150	de	148	e	115	g
Post-R1	162	c	159	cd	154	cde
3wk-R1	190	b	228	a	218	a
PM	193	b	197	b	135	f

‡Pre-V6, soil samples taken 1 week before V6 urea applications; Post-V6, soil samples taken 1 week after V6 urea applications; 3wk-V6, soil samples taken 3 weeks after V6 urea applications; Pre-R1, soil samples taken 1 week before R1 urea application; Post-R1, soil samples taken 1 week after R1 urea applications; 3 wks-R1, soil samples taken 3 weeks after R1 urea applications; PM, soil samples taken at physiological maturity.

§Means within the column followed by different lower case letters are significantly different at the *p ≤* 0.05.

#### Waseca

In 2014, differences started to take place at the 3wk-V6 sampling, where the control tended to have lower tissue N uptake than the treatments receiving N (Table 9). Meaningful differences between treatments started at the 3wk-R1 sampling and continued until the last sampling. During the 3wk-R1 to PM sampling, tissue N uptake was greater when 100% of the recommended rate was applied and decreased as N application rate decreased, especially at the PM sampling. In 2015, although differences started at the Post-V6 sampling, meaningful differences started at the Pre-R1 and remained until the PM sampling. There was a trend for tissue N uptake to be greater when N application was split into two or three applications during the growing season compared with single application. For example, tissue N uptake in the Sp100 and Tsp100 was greater than the PP100 from the Pre-R1 sampling to the last sampling at PM. There was also a clear response to N application rate, where tissue N uptake increased as N application rate increased in the split treatments. In 2016, differences started as soon as the Post-V6 sampling, due to the different rates applied and not due to the time of fertilizer application. From the Post-R1 to PM sampling tissue N uptake tended to be greater in Sp and TSp treatments compared with F treatments. For example, tissue N uptake in the TSp100 was 185 and 277 kg N ha^-1^ at Post-R1 and 3wk-R1, respectively, while tissue N uptake at F100 was 152 and 248 kg N ha^-1^ at Post-R1 and 3wk-R1, respectively.

**Table 9:** Maize tissue N uptake at Waseca 2014–2016.

	2014						
Treatment ‡	Pre-V6†	Post-V6	3wk-V6	Pre-R1	Post-R1	3wk-R1	PM
	kg N ha^-1^						
	4.7	28	63 b§	69 b	68 c	83 d	142 d
F100	5.9	42	93 a	70 b	92 abc	132 abc	180 abc
F125	5.2	32	89 ab	85 ab	110 ab	151 ab	210 a
PP100	4.8	45	93 a	92 ab	121 ab	141 abc	160 bcd
PP125	5.6	35	91 ab	84 ab	132 a	161 a	197 a
Sp75	5.4	33	100 a	96 ab	106 ab	142 abc	162 bcd
Sp100	4.3	35	106 a	106 a	122 ab	144 abc	182 ab
TSp50	4.1	31	83 ab	81 ab	98 abc	110 cd	148 cd
TSp75	4.7	31	92 a	67 b	91 bc	117 bcd	158 bcd
TSp100	4.9	33	103 a	88 ab	127 a	118 bcd	187 ab
	2015						
Treatment	Pre-V6†	Post-V6	3wk-V6	Pre-R1	Post-R1	3wk-R1	PM
	kg N ha^-1^						
C	1.7	13 b	31 c	47 d	67 e	92 d	84 g
F100	5.4	42 a	95 a	129 abc	182 ab	223 ab	230 ab
F125	6.3	40 a	86 ab	129 abc	150 bcd	214 abc	188 cd
PP100	3.3	42 a	62 b	110 bc	116 d	195 bc	156 ef
PP125	5.1	51 a	93 a	137 ab	186 a	225 ab	177 de
Sp75	3.9	36 a	94 a	148 a	162 abc	208 bc	198 bcd
Sp100	3.6	47 a	109 a	153 a	189 a	253 a	220 abc
TSp50	3.6	38 a	60 b	103 c	130 cd	178 c	138 f
TSp75	3.5	31 a	81 ab	128 abc	169 ab	233 ab	209 abcd
TSp100	3.2	46 a	106 a	141 ab	161 abc	254 a	236 a
	2016						
Treatment	Pre-V6†	Post-V6	3wk-V6	Pre-R1	Post-R1	3wk-R1	PM
	kg N ha^-1^						
C	1.8	29 c	25 d	-	75 d	105 e	73 e
F100	3.2	50 ab	54 abc	-	152 bc	248 bcd	126 cd
F125	3.1	61 ab	55 abc	-	151 c	216 d	134 cd
PP100	3.4	82 a	77 a	-	213 a	265 abc	159 abc
PP125	3.5	77 a	69 ab	-	194 a	235 cd	157 abc
Sp75	3.2	56 ab	54 abc	-	152 bc	232 cd	159 abc
Sp100	3.1	66 ab	67 abc	-	192 a	296 a	173 ab
TSp50	1.8	44 bc	40 cd	-	137 c	225 cd	115 d
TSp75	2.8	50 ab	46 bcd	-	154 bc	235 cd	143 bcd
TSp100	3.4	66 ab	62 abc	-	185 ab	277 ab	185 a

**†**Pre-V6, soil samples taken 1 week before V6 urea applications; Post-V6, soil samples taken 1 week after V6 urea applications; 3wk-V6, soil samples taken 3 weeks after V6 urea applications; Pre-R1, soil samples taken 1 week before R1 urea application; Post-R1, soil samples taken 1 week after R1 urea applications; 3 wks-R1, soil samples taken 3 weeks after R1 urea applications; PM, soil samples taken at physiological maturity.

‡C, control no N applied; F100, N applied in the fall at 100% of the recommended rate; F125, N applied in the fall at 125% of the recommended rate; PP100, N applied at pre-plant in the spring at 100% of the recommended rate; PP125, N applied at pre-plant in the spring at 125% of the recommended rate; Sp75, half of the N applied at pre-plant in the spring and half at V6 at 75% of the recommended rate; Sp100, half of the N applied at pre-plant in the spring and half at V6 at 100% of the recommended rate; TSp50, a third of the N applied at pre-plant in the spring, a third at V6 and a third at R1 at 50% of the recommended rate; TSp75, a third of the N applied at pre-plant in the spring, a third at V6 and a third at R1 at 75% of the recommended rate; TSp100, a third of the N applied at pre-plant in the spring, a third at V6 and a third at R1 at 100% of the recommended rate.

§Means within the column followed by different lower case letters are significantly different at *p ≤* 0.05.

In general, there was a decrease in tissue N uptake at all locations from the 3wk-R1 to the PM sampling, which could be due to the fact that by this stage a significant amount of N had been translocated from the plant tissue to the developing ear. The variation observed in tissue N uptake at Becker, Lamberton and Waseca confirms the significance of environment in maize production systems as noted by Sindelar et al. [31]. At Becker and Waseca, the influence of time of application on tissue N uptake is demonstrated with the C, PP, and F treatments having lower tissue N uptake than Sp and TSP treatments applied at agronomically optimum N rates. In contrast, at Lamberton, tissue N uptake in Sp and TSp was as effective as F and PP treatments. The response of maize tissue N uptake to N rate, particularly at Lamberton and Waseca, supports the conclusion that total aboveground maize N uptake responds to increasing rates of N fertilizer [19]. Similar results were also reported by Halvorson and Jantalia [32], where stover yields increased with increasing N rates.

### Recovery efficiency

#### Becker

The timing of fertilizer application had an impact on RE at Becker. The TSp50 treatment recovered 0.60 kg kg^-1^ of applied fertilizer, more than all other treatments except TSp100 (Table 10). There was no difference in RE between Sp and TSp treatments receiving at least 75% of recommended N fertilizer rates. All TSp treatments, regardless of rate, were greater than the F and PP treatments while Sp75 and Sp100 were greater than the F treatments. Recovery efficiency increased by 6 to 8% annually from 0.24 kg kg^-1^ in 2014 to 0.38 kg kg^-1^ in 2016.

**Table 10:** Recovery efficiency of applied N fertilizer as affected by treatment and location, averaged across years.

Treatment†	Becker		Lamberton		Waseca	
	kg kg^-1^					
C						
F100	0.068	d‡	0.658	a	0.424	abcd
F125	0.112	d	0.614	a	0.358	bcd
PP100	0.209	cd	0.647	a	0.308	d
PP125	0.221	cd	0.557	a	0.344	cd
Sp75	0.357	bc	0.656	a	0.508	ab
Sp100	0.344	bc	0.636	a	0.493	abc
TSp50	0.601	a	0.619	a	0.353	bcd
TSp75	0.402	b	0.628	a	0.485	abc
TSp100	0.462	ab	0.632	a	0.548	a

†C, control no N applied; F100, N applied in the fall at 100% of the recommended rate; F125, N applied in the fall at 125% of the recommended rate; PP100, N applied at preplant in the spring at 100% of the recommended rate; PP125, N applied at preplant in the spring at 125% of the recommended rate; Sp75, half of the N applied at preplant in the spring and half at V6 at 75% of the recommended rate; Sp100, half of the N applied at preplant in the spring and half at V6 at 100% of the recommended rate; TSp50, a third of the N applied at preplant in the spring, a third at V6 and a third at R1 at 50% of the recommended rate; TSp75, a third of the N applied at preplant in the spring, a third at V6 and a third at R1 at 75% of the recommended rate; TSp100, a third of the N applied at preplant in the spring, a third at V6 and a third at R1 at 100% of the recommended rate.

‡Means within the column followed by different low case letters are significantly different at the *p ≤* 0.05.

#### Lamberton

Over the three study years there was no difference in RE between treatments (Table 10). This could be due to high N mineralization in the Normania loam soil. The average RE was 0.78 kg kg^-1^ in 2014, 0.68 kg kg^-1^ in 2015 and 0.42 kg kg^-1^ in 2016. The RE of TSp50, TSp75 and Sp75 averaged 0.63 kg kg-1 comparable to F and PP treatments.

#### Waseca

Across 2014 to 2016, TSp100 had a RE of 0.55 kg kg^-1^, greater than TSp50, F125, PP100, and PP125 which ranged from 0.31 to 0.36 kg kg^-1^ (Table 10). Split applications receiving at least 75% recommended rates recovered an average 0.51 kg kg^-1^ of applied fertilizer compared with F and PP treatments which averaged a RE of 0.36 kg kg^-1^. The reduced RE of F treatments compared with Sp and TSp treatments across all three study years suggest that applying N fertilizer as a split application can improve nitrogen use efficiency.

Across locations, there was no difference in RE between treatments in 2014 (Table 11). However, in 2015 TSp75 had a RE of 0.71 kg kg^-1^, greater than all other treatments except TSp100 and Sp75. Preplant applications had an average RE of 0.368 kg kg^-1^, lower than all other treatments except F125. There was less difference between treatments in 2016; however, TSp100 and TSp50 produced greater RE than F100, F125, and PP125 (Table 11). F100 had a lower RE than all treatments except F125 and PP125.

**Table 11:** Recovery efficiency of applied N fertilizer as affected by treatment and year, averaged across locations.

Treatment †	2014		2015		2016	
	kg kg^-1^					
C						
F100	0.433	a‡	0.504	bc	0.213	d
F125	0.403	a	0.423	cd	0.258	cd
PP100	0.383	a	0.363	d	0.418	abc
PP125	0.427	a	0.373	d	0.323	bcd
Sp75	0.388	a	0.644	ab	0.488	ab
Sp100	0.431	a	0.561	bc	0.482	ab
TSp50	0.468	a	0.543	bc	0.562	a
TSp75	0.386	a	0.707	a	0.423	abc
TSp100	0.496	a	0.643	ab	0.503	a

† C, control no N applied; F100, N applied in the fall at 100% of the recommended rate; F125, N applied in the fall at 125% of the recommended rate; PP100, N applied at preplant in the spring at 100% of the recommended rate; PP125, N applied at preplant in the spring at 125% of the recommended rate; Sp75, half of the N applied at preplant in the spring and half at V6 at 75% of the recommended rate; Sp100, half of the N applied at preplant in the spring and half at V6 at 100% of the recommended rate; TSp50, a third of the N applied at preplant in the spring, a third at V6 and a third at R1 at 50% of the recommended rate; TSp75, a third of the N applied at preplant in the spring, a third at V6 and a third at R1 at 75% of the recommended rate; TSp100, a third of the N applied at preplant in the spring, a third at V6 and a third at R1 at 100% of the recommended rate.

‡Means within the column followed by different low case letters are significantly different at *p ≤* 0.05.

## Discussion

### Becker

On coarse-textured soils, split applications of N fertilizer, particularly TSp, improved maize grain yield, N uptake, AE, and RE compared with a single application in the fall or just before planting. Conversely, applying N in the fall produced low grain yields, N uptake and NUE. That the soil cannot retain N over the period in the year when high water infiltration rates are expected, highlights the importance of synchronizing N application with crop needs. The maize grain yield results from Becker corroborate findings from another study on coarse-textured soils where a split application of 185 kg N ha^-1^ produced maize grain yield that was equivalent or greater than that with 250 to 300 kg N ha^-1^ as a single preplant application [33]. Similarly, on irrigated sandy soils, Rubin et al. [6] reported that split application (preplant and at the four leaf-collar stage) of urea increased maize grain yield by 5.4% compared with a single preplant application of enhanced efficiency N fertilizers. The AE achieved when using a split application demonstrates the potential for improved NUE in sandy soils. With three years of consistently greater AE compared with F or PP treatments, the results confirm that the most efficient BMP for irrigated sandy soils is the use of split N application [34]. However, the annual increase in RE at Becker along with increased fertilizer rate disagrees with the typical trend of RE to decrease as the amount of N-fertilizer applied increases [12].

### Lamberton

The lack of difference in maize grain yield, N uptake, AE, and RE among treatments applied at recommended rates suggests that Normania loam soils are not favorable for N loss. The minimization of N loss could be a reason why maize performs well under low N rates. The lack of differences among treatments applied in the fall, at planting and as a split application are similar to findings by Venterea and Coulter [14] and Venterea et al. [8] which showed no difference between single and split N applications on non-irrigated silt loam soils in the upper Midwest. However, the lack of difference in yield between Sp75 and F125 in 2014 could be due to soil N mineralization. May 2014 was relatively dry while June received 188 mm of precipitation, 84 mm more than the 30-yr average, and precipitation in July was only 30 mm (Table 1). It is likely that the abundant precipitation in June improved soil N mineralization while facilitating rapid maize uptake of N applied at V6. In contrast, dry conditions during July could have hindered maize uptake of N applied at silking for the TSp treatments. In contrast to Becker, AE at Lamberton was influenced more by rate of N application than timing of N application (Table 5). Across the three years, AE tended to decrease as the rate of N fertilizer increased, similar with results from Sindelar et al. [19]. However, the RE at Lamberton was similar to the RE of 67% recorded by Wortmann et al. [17] using EONR in Nebraska. These findings corroborate findings by Venterea et al. [8] who also did not find any difference between split or single applied treatments in a rainfed system. However, the results could also demonstrate that slightly reducing the N rate and applying N as a split application could improve RE and NUE.

### Waseca

At Waseca, a Nicollet clay loam soil, the year-to-year trend suggested that split applications when applied at recommended rates tended to maximize grain yields, N uptake, and NUE parameters. With TSp75 yielding approximately 5% less grain compared with treatments receiving recommended rates, demonstrates that split applications in fine textured soils can enable a reduction in N fertilizer rate without reductions in grain yield, thereby enhancing efficiency of applied N and reducing risk of negative environmental impacts. Similar results were observed in a study where there were no differences in yield between fertilizer applied at 100 and 85% recommended rates [8]. The wet June in 2014 likely enhanced loss of N applied at planting and at the V6 stage while also restricting root development and lowering yield. Furthermore, N could have been lost through denitrification as fine-textured soils, especially ones characterized by low permeability, can lose N via denitrification in as early as a few days if N fertilization is followed by the establishment of waterlogged conditions [35]. Abundant and well-distributed precipitation during July through September of 2016 could have enhanced maize utilization of N applied at V6 and silking (Table 1). The lower N application in TSp50 likely resulted in less leaching potential that did not impact soil N content. This was demonstrated by modeled findings by Gowda et al. [36] from a 365-ha watershed in the upper Midwest where nitrate-N losses were reduced by 21 kg ha^-1^ (17%) when there was a 20% reduction in spring-applied fertilizer rate. At Waseca maximum grain yield in all three study years was achieved with Sp100 and PP125. This supports the conclusion of the modeled annual findings of Randall et al. [15] where maize grain yield with split application of N (40% preplant and 60% at the eight leaf-collar stage of maize phenological development) was greater than that with fall or preplant application of N. Vetsch and Randall [3] reported that when wet and warm spring conditions followed a late fall N application, maize grain yield and aboveground N uptake decreased by 20 and 27%, respectively. In the present study, average air temperature in April, May and June 2016 was 1.3, 0.3, and 1.2C° warmer than historical averages, supporting this observation. The lack of differences in AE among PP100, Sp100, and TSp100 is similar to conclusions by Wortmann et al. [17] where the application of N near to economic optimum nitrogen rates improved NUE. The low average recovery efficiency of PP treatments (0.368 kg kg^-1^) was likely due to the wet May, June, and July in 2015. N fertilizer applied as PP likely leached before it could be taken up, whereas the split applications, with an average RE of 0.62 kg kg^-1^, benefited from N mineralization and a more established root structure to better facilitate N uptake (Tables 1 and 11). Similarly, the wet July and August in 2016 likely improved N mineralization and maize growth, resulting in improved RE in split application treatments. Overall, with TSp75 yielding approximately 5% less grain compared with treatments receiving recommended rates, demonstrate that split applications in fine textured soils can enable a reduction in N fertilizer rate without reductions in grain yield, thereby enhancing efficiency of applied N and reducing risk of negative environmental impacts.

## Conclusions

There is a clear need for site-specific N fertilizer management to optimize maize grain yield and improve NUE across different soils and growing environments. Further study on whether there is an economic benefit to using split N applications along with determining any long-term risks will further assist growers. If the weather pattern observed during this study of warmer fall and wetter spring conditions continue there may be greater need for research to better integrate weather forecasts with soil N supply to maintain high maize yield while reducing negative environmental impacts from N fertilizer.

## Abbreviations

AE: agronomic efficiency
BMP: best management practice
F: fall application
N: nitrogen
NUE: nitrogen use efficiency
PP: preplant application
RE: recovery efficiency
RN: recommended nitrogen rate
R1: silking stage of maize phenological development
Sp: two-way split application of nitrogen
TSp: three-way split application of nitrogen
V6: six leaf-collar stage of maize phenological development
